# Melatonin protects mesenchymal stem cells from autophagy‐mediated death under ischaemic ER‐stress conditions by increasing prion protein expression

**DOI:** 10.1111/cpr.12545

**Published:** 2018-11-14

**Authors:** Jun Hee Lee, Yeo Min Yoon, Yong‐Seok Han, Seo Kyung Jung, Sang Hun Lee

**Affiliations:** ^1^ Department of Pharmacology and Toxicology University of Alabama at Birmingham School of Medicine Birmingham Alabama; ^2^ Medical Science Research Institute Soonchunhyang University Seoul Hospital Seoul Korea; ^3^ Departments of Biochemistry Soonchunhyang University College of Medicine Cheonan Korea

**Keywords:** autophagy, cellular prion protein, endoplasmic reticulum stress, melatonin, mesenchymal stem cell

## Abstract

**Object:**

The purpose of this study was to explore whether melatonin could protect mesenchymal stem cells (MSCs) against ischaemic injury, by inhibiting endoplasmic reticulum (ER) stress and autophagy both in vivo and in vitro.

**Materials and Methods:**

To confirm the protective effect of melatonin against ER stress in MSCs, markers of cell viability, apoptosis and autophagy were analysed. To further investigate the regenerative effect of melatonin‐treated MSCs in ischaemic tissues, a murine hindlimb ischaemic model was established.

**Results:**

Under oxidative stress conditions, treatment with melatonin suppressed the activation of ER stress–associated proteins and autophagy‐associated proteins acting through upregulation of cellular prion protein (PrP^C^) expression. Consequently, inhibition of apoptotic cell death occurred. Melatonin also promoted the activation of MnSOD and catalase activities in MSCs. In a murine hindlimb ischaemia model, melatonin‐treated MSCs also enhanced the functional limb recovery as well as neovascularization. These beneficial effects of melatonin were all blocked by knock‐down of PrP^C^ expression.

**Conclusion:**

Melatonin protects against ER stress/autophagy‐induced apoptotic cell death by augmenting PrP^C^ expression. Thus, melatonin‐treated MSCs could be a potential cell‐based therapeutic agent for ER stress–induced ischaemic diseases, and melatonin‐induced PrP^C^ might be a key molecule in ameliorating ER stress and autophagy.

## 1 INTRODUCTION

Mesenchymal stem cells (MSCs) represent one type of stem/progenitor cell‐based therapies used to treat ischaemic diseases, such as stroke, cardiovascular disease, peripheral arterial disease, and ischaemic kidney disease.^1^ Since these cells have a self‐renewal ability, multi‐differentiation potential, and an immunomodulatory effect, various studies have investigated the potential benefit of MSC‐based therapies in pre‐clinical and clinical trials.^1^, ^2^ Unfortunately, transplanted MSCs have shown a low survival rate in ischaemic‐damaged tissue because a sudden or prolonged blockage of blood flow induces hypoxia, oxidative stress, endoplasmic reticulum (ER) stress and nutrient deprivation.^3^, ^4^ To improve the therapeutic efficacy of MSC‐based therapies in pathophysiological conditions, a number of different approaches have been explored, including preconditioning, genetic modification, co‐transplantation of MSCs with other supporting cells, administration of MSCs along with medication and application of the MSCs with biomaterials.^5^ Although these strategies have improved the functionalities of MSCs, it is important to understand the underlying mechanisms behind these strategies in order to safely use MSC‐based therapies in ischaemic diseases. The ER is an important organelle for protein folding, calcium homoeostasis, and lipid and carbohydrate metabolism.^6^ Endoplasmic reticulum stress occurs in response to a range of cellular stresses, such as ischaemic damage, redox imbalance, oxygen and glucose deprivation and perturbations in calcium homoeostasis.^6^, ^7^ To maintain cellular homoeostasis against ER stress, cells have well‐orchestrated processes, including ER‐assisted degradation, autophagy, the hypoxia response and mitochondrial biogenesis.^6^, ^8^ Among these processes, autophagy plays a pivotal role in cell survival, cell growth, energy metabolism and lifespan in several cellular stresses under physiological conditions.^6^, ^8^ However, under pathophysiological conditions, severe stress induces excessive autophagy, causing cell death.^9^ Therefore, in order to create a successful cell‐based therapy for ischaemic diseases, the key is to inhibit the excessive autophagy without eliminating the basal autophagy level.

The normal cellular prion protein (PrP^C^) is encoded by the *Prnp* gene, which plays a physiological role in various cells.^10^ Accumulating evidence indicates roles for PrP^C^ in a wide range of cellular processes, such as stress protection, oxidative stress‐induced apoptosis, the ER‐stress response, cellular differentiation, the immune system, mitochondrial homoeostasis, and metal ion homoeostasis.^11^ Knockout of the *Prnp* gene induces a dysfunction in synaptic transmission and plasticity, memory formation, calcium homoeostasis, neurite outgrowth and metabolism.^10^ In stem cell biology, PrP^C^ is essential for cell proliferation, differentiation and self‐renewal.^12^, ^13^, ^14^ In addition, our previous studies have shown that PrP^C^ is up‐regulated by hypoxia preconditioning and melatonin treatment, and that transplanted MSCs facilitate neovascularization in a murine hindlimb ischaemia model.^15^, ^16^ Moreover, augmentation of PrP^C^ expression by treatment of MSCs with tauroursodeoxycholic acid inhibited ER stress–mediated cell death.^17^ Therefore, an understanding of the role of PrP^C^ may provide insights into the development of stem/progenitor cell‐based therapies.

Melatonin, N‐acetyl‐5‐methoxytryptamine, is an indoleamine containing hormone secreted by several tissues including the bone marrow, ovary, testes, gut, placenta and liver, as well as the pineal gland.^18^, ^19^ Melatonin has a variety of functions, including the regulation of sleep and circadian rhythms, as well as having anti‐inflammatory, antioxidant and anti‐cancer effects.^20^, ^21^, ^22^, ^23^ Numerous studies have shown that melatonin‐treated MSCs can facilitate a therapeutically functional recovery in myocardial infarction, skin wounds, lung ischaemia‐reperfusion injury and sepsis‐induced kidney injury.^24^, ^25^, ^26^, ^27^ In addition, our previous study has shown that melatonin‐treated MSCs enhance neovascularization in hindlimb ischaemia through PrP^C^ expression.^16^ This study, as an extension of this research, focuses on the effect of melatonin on ER stress in ischaemic disease. We show here that melatonin protects against autophagy‐mediated apoptosis both in vitro and in vivo following ischaemic‐induced ER stress and this occurs through the upregulation of PrP^C^ expression. In addition, we demonstrate the therapeutic effect of melatonin‐treated MSCs on neovascularization in a murine hindlimb ischaemia model.

## 2 MATERIAL AND METHODS

### 2.1 Cell culture

Human adipose‐derived MSCs were obtained from the American Type Culture Collection (ATCC, Manassas, VA, USA). The supplier certified that the MSCs expressed the cell surface markers (CD73 and CD105) and could undergo adipogenic or osteogenic differentiation when cultured with the appropriate differentiation medium. MSCs were maintained in alpha‐minimum essential medium (Hyclone, Logan, UT, USA) supplemented with 10% (v/v) foetal bovine serum (Hyclone) and 100 U/mL penicillin/streptomycin (Thermo Fisher Scientific, Waltham, MA, USA). MSCs were incubated in a humidified incubator at 37°C and 5% CO_2_.

### 2.2 Chemical treatment of MSCs

Mesenchymal stem cells were washed twice with phosphate‐buffered saline (PBS), and suspended in fresh α‐MEM supplemented with 10% FBS. To detect the activation of the apoptotic signalling pathway, MSCs were pretreated with melatonin (1 µM) at 37°C for 30 min and then treated with H_2_O_2_ (200 µM) for the indicated durations (0, 2, 4, or 8 hours).

### 2.3 Animal care procedures and experiments

The Institutional Animal Care and Use Committee of Soonchunhyang University approved all surgical interventions and post‐operative animal care (IACIC2013‐5), and these were performed in accordance with the National Research Council Guidelines for the Care and Use of Laboratory Animals. Animal experiments were performed on 8‐week‐old male Balb/C nude mice (Biogenomics, Seoul, Korea), which were maintained in a pathogen‐free facility under a 12‐hours light/dark cycle at 25°C with free access to water and regular laboratory chow in accordance with the regulations of Soonchunhyang University, Seoul Hospital.

### 2.4 Murine hindlimb ischaemia model

Experiments using a murine hindlimb ischaemia model were performed as previously reported with minor modifications.^15^, ^28^ Ischaemia was induced by the ligation and excision of the proximal femoral artery and the boundary vessels of the mice. No later than 6 hours after surgery, PBS, untreated MSCs, or melatonin‐treated MSCs transfected with *PRNP* siRNA or a scrambled siRNA were injected intramuscularly into the ischaemic thigh area (5 × 10^5^ cells/100 μL PBS per mouse; five mice per treatment group). Each mouse was injected with cells (or PBS) at five ischaemic sites. Thereafter, melatonin (20 mg/kg/day) was administered intraperitoneally daily for 28 days. The melatonin stock solution was diluted in water, so that the final ethanol concentration was below 1%. Blood perfusion was assessed by measuring the ratio of the blood flow in the ischaemic (left) limb to that in the non‐ischaemic (right) limb on post‐operative days 0 and 28 using laser Doppler perfusion imaging (LDPI; Moor Instruments, Wilmington, DE, USA).

### 2.5 Statistical analysis

All data are expressed as the mean ± *SE* of the mean (SEM) and all of the experiments were analysed using a one‐way analysis of variance (ANOVA). Some comparisons of ≥3 groups were made using the Bonferroni‐Dunn test. A *P* value <0.05 was considered statistically significant. These data were statistically analysed using SigmaPlot (Systat Software, San Jose, CA, USA).

Detailed some methods are provided in Appendix [Link].

## 3 RESULTS

### 3.1 Ischaemia‐mediated ROS induces apoptosis through ER stress in MSCs and in hindlimb ischaemic‐injured tissues

**Figure 1:**
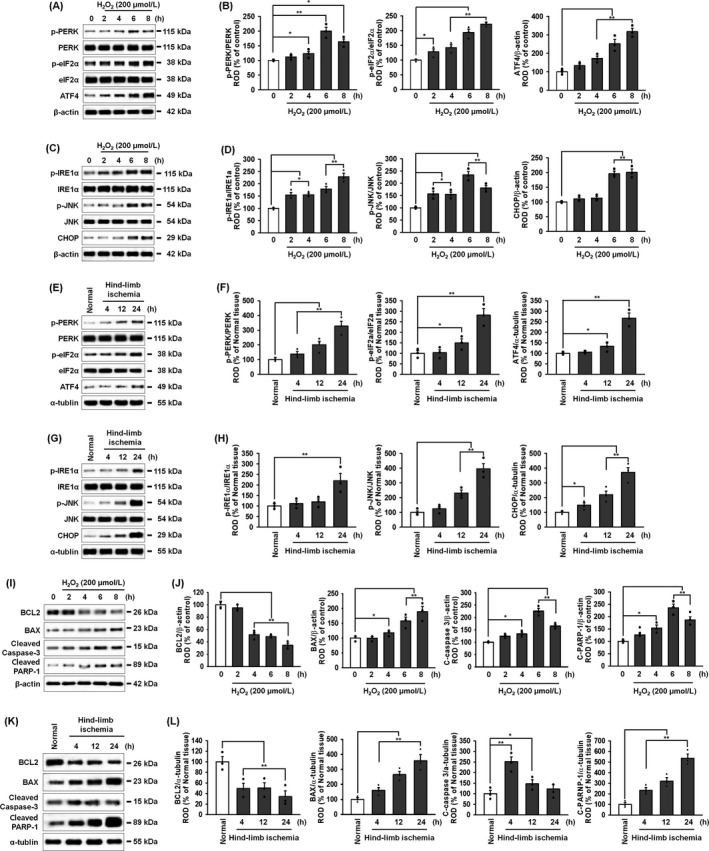
Endoplasmic reticulum stress–mediated apoptotic cell death in MSCs and ischaemic tissue. A, Western blot of p‐PERK, PERK, p‐eIF2α, eIF2α and ATF4 after treatment of MSCs with hydrogen peroxide (200 μmol/L) for 0, 2, 4, 6 and 8 hours. B, The levels of p‐PERK, p‐eIF2α and ATF4 were normalized to those of PERK, eIF2α and β‐actin, respectively. Values represent the mean ± SEM. *P < 0.05; **P < 0.01 vs untreated MSCs. C, Western blot of p‐IRE1α, IRE1α, p‐JNK, JNK and CHOP after treatment of MSCs with hydrogen peroxide (200 μmol/L) for 0, 2, 4, 6 and 8 hours. D, The levels of p‐IRE1α, p‐JNK and CHOP were normalized to those of IRE1α, JNK and β‐actin, respectively. Values represent the mean ± SEM. *P < 0.05; **P < 0.01 vs untreated MSCs. E, Western blot of p‐PERK, PERK, p‐eIF2α, eIF2α and ATF4 in ischaemic‐injured tissues from the murine hindlimb ischaemia model, 4, 12 and 24 hours after surgery. F, The expression of p‐PERK, p‐eIF2α and ATF4 were normalized to those of PERK, eIF2α and α‐tubulin, respectively. Values represent the mean ± SEM. *P < 0.05; **P < 0.01 vs normal tissue. G, Western blot of p‐IRE1α, IRE1α, p‐JNK, JNK and CHOP in ischaemic‐injured tissues from the murine hindlimb ischaemia model, 4, 12 and 24 hours after surgery. H, The expression of p‐IRE1α, p‐JNK and CHOP were normalized to those of IRE1α, JNK and α‐tubulin, respectively. Values represent the mean ± SEM. *P < 0.05; **P < 0.01 vs normal tissue. I, Western blot of BCL2, BAX, cleaved caspase‐3, and cleaved PARP‐1 after treatment of MSCs with hydrogen peroxide (200 μmol/L) for 0, 2, 4, 6 and 8 hours. J, All protein expression levels were normalized to that of β‐actin. Values represent the mean ± SEM. *P < 0.05; **P < 0.01 vs untreated MSCs. K, Western blot of BCL2, BAX, cleaved caspase‐3 and cleaved PARP‐1 in ischaemic‐injured tissues from the murine hindlimb ischaemia model, 4, 12 and 24 hours after surgery. L, All protein expression levels were normalized to that of α‐tubulin. Values represent the mean ± SEM. *P < 0.05; **P < 0.01 vs normal tissue

ROS is one of the inducers of ER stress which is known to be involved in cell life and death.^29^ Our previous study demonstrated that ischaemia‐induced ROS‐mediated ER stress–triggered apoptosis in ischaemic tissues and led to a deterioration in the quality and efficacy of the transplanted MSCs used as a cell‐based therapy for ischaemic diseases.^17^ To further investigate the effect of ROS on ER stress in MSCs and ischaemic tissues, the activation and expression of ER stress–associated proteins was assessed in MSCs treated with H_2_O_2_ (Figure 1)_,_ as well as in ischaemic‐injured tissues in a murine hindlimb ischaemia model. In MSCs, H_2_O_2,_ as an ER‐stress inducer, increased the activation and expression of these ER stress–associated proteins (Figure 1A–D). In addition, the activation and expression of these proteins were also increased in ischaemic‐injured tissues (Figure 1E–H). These data indicate that ischaemia‐induced ROS induces the activation and expression of ER stress–mediated proteins. Furthermore, we assessed the level of apoptosis‐associated proteins in MSCs and in ischaemic tissues under these same ROS‐mediated ER‐stress conditions (Figure 1I–L). After treatment of MSCs with H_2_O_2_, the level of the anti‐apoptotic protein, BCL2 decreased and the expression of pro‐apoptotic proteins, including BAX, cleaved caspase‐3 and cleaved PARP‐1 increased. In ischaemic tissues, similar apoptosis expression level with treatment MSCs with H_2_O_2_. These results indicate that ischaemia‐mediated ROS leads to apoptosis via ER stress in MSCs and in hindlimb ischaemic‐injured tissues.

### 3.2 ROS‐mediated ER stress activates autophagy in both MSCs and hindlimb ischaemic‐injured tissues

**Figure 2:**
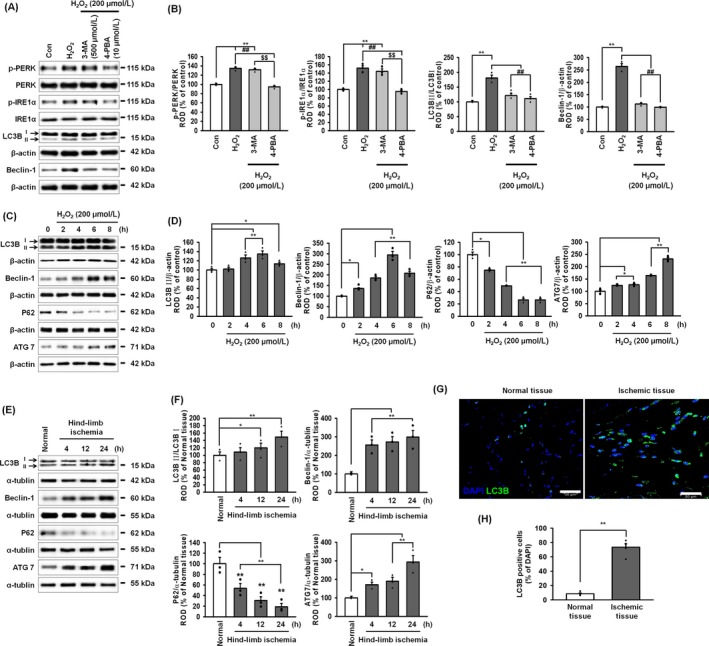
ER stress–mediated autophagy in MSCs and ischaemic tissue. A, Western blot of p‐PERK, PERK, p‐IRE1α, IRE1α, LC3B and Beclin‐1 after pre‐treatment of MSCs with 3‐MA (500 μmol/L; an autophagy inhibitor) or 4‐PBA (10 μmol/L; an ER‐stress inhibitor) followed by treatment with hydrogen peroxide (200 μmol/L). B, The levels of p‐PERK, p‐eIF2α, LC3BII and Beclin‐1 were normalized to those of PERK, eIF2α, LC3BI and β‐actin, respectively. Values represent the mean ± SEM. **P < 0.01 vs untreated MSCs, ^##^ *P* < 0.01 vs MSCs treated with H_2_O_2_, and ^$^ *P* < 0.05; ^$$^ *P* < 0.01 vs 3‐MA‐pretreated MSCs treated with H_2_O_2_. C, Western blot of LC3B, Beclin‐1, p62 and ATG7 in cells after treatment of MSCs with hydrogen peroxide (200 μmol/L) for 0, 2, 4, 6 and 8 hours. D, The levels of LC3BII were normalized to LC3B1 whereas the levels of Beclin‐1, p62, and ATG7 were normalized to β‐actin. Values represent the mean ± SEM. *P < 0.05; **P < 0.01 vs untreated MSCs. E, Western blot of LC3B, Beclin‐1, p62, and ATG7 in ischaemic‐injured tissues from the murine hindlimb ischaemia model, 4, 12 and 24 hours after surgery. F, The levels of LC3BII were normalized to LC3B1 whereas the levels of Beclin‐1, p62 and ATG7 were normalized to α‐tubulin. Values represent the mean ± SEM. *P < 0.05; **P < 0.01 vs normal tissue. G, Immunofluorescent staining for DAPI (blue) and LC3B (green) in normal and ischaemic tissues. Scale bar = 30 μm. H, Autophagic cells were quantified as the percentage of LC3B‐positive cells per total DAPI‐stained cells. Values represent the mean ± SEM. **P < 0.01 vs normal tissue

Under physiological conditions, ER stress induces autophagy in order to remove misfolded proteins, resulting in the inhibition of cell death; however, under pathophysiological conditions, severe autophagy leads to cell death.^30^ To evaluate the effect of ROS‐mediated ER stress on autophagy in MSCs, the expression of autophagy marker proteins was examined after treatment of the MSCs with H_2_O_2_. Treatment with H_2_O_2_ increased activation of the ER‐stress proteins, PERK and IRE1α, but the autophagy inhibitor 3‐MA (500 μmol/L) did not significantly decrease this H_2_O_2_‐induced activation of these proteins (Figure 2A,B). ROS‐mediated ER stress also increased the expression of the autophagy markers, LC3B and Beclin‐1, and 4‐PBA (10 μmol/L) an ER‐stress inhibitor, suppressed this ROS‐induced augmentation of autophagy marker expression (Figure 2A,B). In addition, ROS‐mediated ER stress increased the expression of another autophagy marker, ATG7, as well as LC3B and Beclin‐1, and decreased the level of p62, which is known to be decreased following autophagy^31^ (Figure 2C,D). These findings indicate that autophagy is activated following ROS‐mediated ER stress. To further examine the effect of ischaemic‐induced ER stress on autophagy in ischaemic‐injured tissues, the expression of LC3B, Beclin‐1, and ATG7 were increased and the level of p62 was decreased (Figure 2E,F). In addition, immunofluorescence staining for LC3 showed that the level of LC3 was significantly increased in ischaemic tissue, compared with that in normal tissue (Figure 2G,H). Taken together, these results suggest that ischaemia‐induced ER stress activates the autophagy process.

### 3.3 Melatonin protects cells from oxidative stress through the melatonin‐MT_2_‐PrP^C^ axis

**Figure 3:**
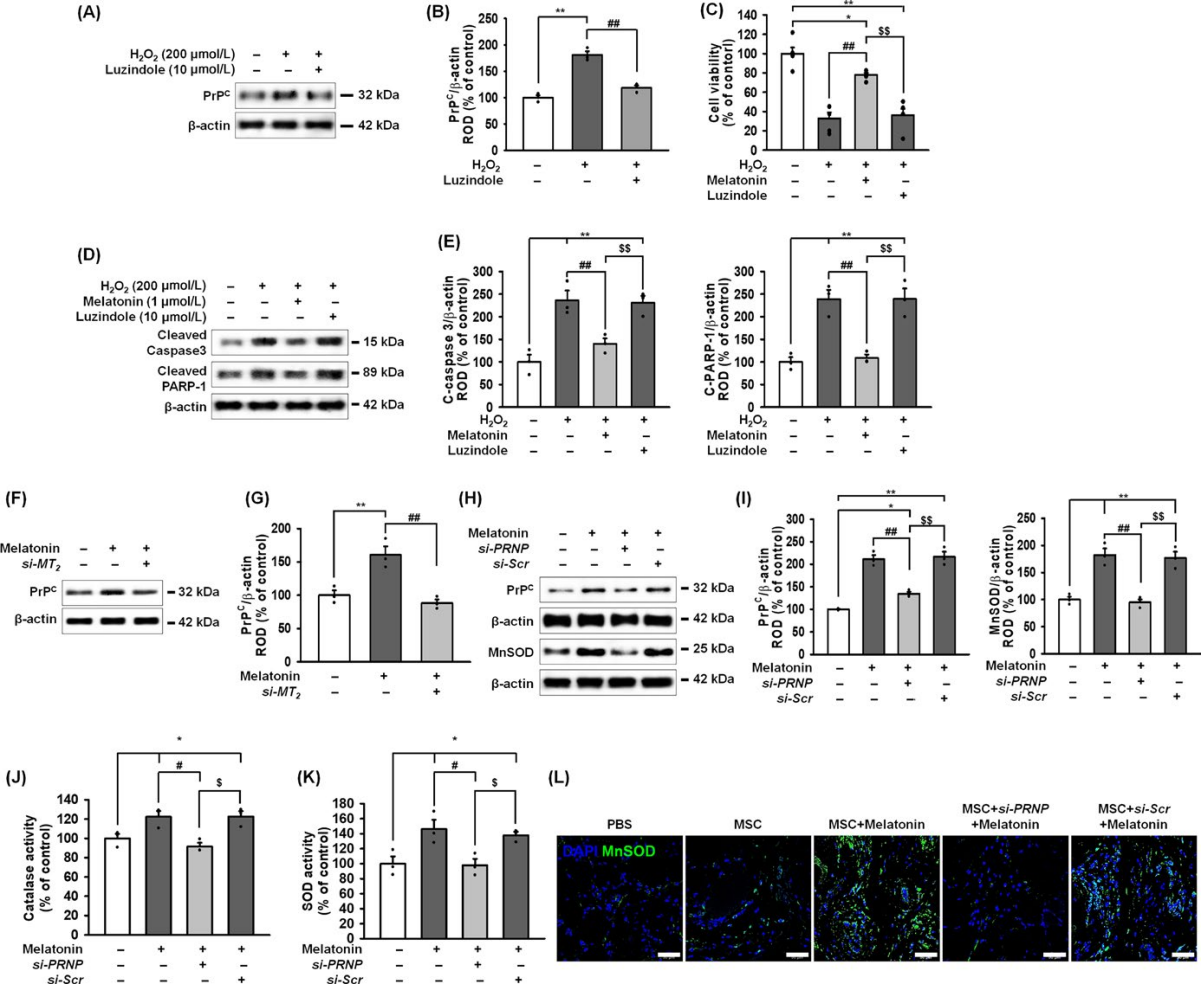
The anti‐oxidative effect of melatonin on MnSOD and catalase reduced cell viability through the melatonin‐MT_2_‐PrP^C^ axis under oxidative stress. A, Expression of PrP^C^ after treatment of MSCs with melatonin and a combination of melatonin and luzindole (10 μmol/L; an MT_1_ and MT_2_ antagonist). B, PrP^C^ was quantified by densitometry and normalized to that of β‐actin. Values represent the mean ± SEM. **P < 0.01 vs untreated MSCs and ^##^ *P* < 0.01 vs MSCs treated with melatonin. C, Viability of melatonin‐treated MSCs after treatment with H_2_O_2_ with/without luzindole. Values represent the mean ± SEM. *P < 0.05; **P < 0.01 vs untreated MSCs and ^##^ *P* < 0.01 vs MSCs +H_2_O_2_. ^$$^ *P* < 0.01 vs MSCs + melatonin + H_2_O_2_. D, Expression of cleaved caspase‐3 and cleaved PARP‐1 after treatment of melatonin‐pretreated MSCs with H_2_O_2_ (200 μmol/L). E, The levels of these proteins were normalized to that of β‐actin. Values represent the mean ± SEM. **P < 0.01 vs untreated MSCs, ^##^ *P* < 0.01 vs MSCs + H_2_O_2_, and ^$$^ *P* < 0.01 vs MSCs + melatonin + H_2_O_2_. F, Western blot of PrP^C^ after treatment of MSCs with melatonin and a combination of melatonin and *MT2 siRNA* (*si‐MT_2_*). G, The level of PrP^C^ was quantified by densitometry and normalized relative to the β‐actin expression level. Values represent the mean ± SEM. **P < 0.01 vs untreated MSCs and ^##^ *P* < 0.01 vs MSCs treated with melatonin. H, Western blot of PrP^C^ and MnSOD after treatment of MSCs with melatonin, a combination of melatonin and *PRNP siRNA* (*si‐PRNP*), or a combination of melatonin and scrambled *siRNA* (*si‐Scr*). I, The expression of PrP^C^ and MnSOD were quantified by densitometry and normalized relative to the β‐actin expression level. Values represent the mean ± SEM. **P < 0.01 vs untreated MSCs, ^##^ *P* < 0.01 vs MSCs treated with melatonin, and ^$$^ *P* < 0.01 vs MSCs treated with melatonin and *si‐PRNP*. J, Catalase activity after treatment of MSCs with melatonin, a combination of melatonin and *si‐PRNP*, or a combination of melatonin and *si‐Scr*. Values represent the mean ± SEM. *P < 0.05 vs untreated MSCs, ^#^ *P* < 0.05 vs MSCs treated with melatonin, and ^$^ *P* < 0.05 vs MSCs treated with melatonin and *si‐PRNP*. K, SOD activity after treatment of MSCs with melatonin, a combination of melatonin and *si‐PRNP*, or a combination of melatonin and *si‐Scr*. Values represent the mean ± SEM. *P < 0.05 vs untreated MSCs, ^#^ *P* < 0.05 vs MSCs treated with melatonin, and ^$^ *P* < 0.05 vs MSCs treated with melatonin and *si‐PRNP*. L, Immunofluorescent staining for DAPI (blue) and MnSOD (green) in ischaemic‐injured tissues injected with PBS, MSCs, melatonin‐treated MSCs, melatonin‐treated MSCs plus *si‐PRNP* or melatonin‐treated MSCs plus *si‐Scr*. Scale bar = 50 μm

Our previous study demonstrated that melatonin enhances MSC bioactivities, including proliferation, anti‐oxidative effects and immunomodulatory effects, through the upregulation of PrP^C^ expression.^16^ Expression of the melatonin receptor 2 (MT_2_) gene is higher than that of MT_1_ in MSCs, and melatonin significantly upregulates MT_2_.^16^ To further investigate whether melatonin regulates the activity of ROS scavenger enzymes via the MT_2_‐mediated expression of PrP^C^, we first confirmed the anti‐oxidative effect of melatonin in MSCs. Treatment of MSCs with H_2_O_2_ significantly increased PrP^C^ levels, whereas treatment with luzindole, a melatonin receptor antagonist that preferentially targets MT_2_ over MT_1_, blocked the increase in PrP^C^ (Figure 3A,B). Under oxidative stress, melatonin increased cell viability, but inhibition of the melatonin receptor significantly decreased the survival of melatonin‐treated MSCs (Figure 3C). Furthermore, melatonin significantly inhibited the expression of cleaved caspase‐3 and cleaved PARP‐1 under oxidative stress, whereas this protective effect was blocked by luzindole (Figure 3D,E). These results indicate that melatonin protects MSCs from oxidative stress through the melatonin receptor.

To further investigate the protective effect of melatonin on oxidative stress via the MT_2_‐PrP^C^ axis, we knocked‐down MT_2_ in MSCs and analysed the anti‐oxidative effect of melatonin. Treatment of MSCs with melatonin significantly increased PrP^C^ expression and knock‐down of MT_2_ blocked this melatonin‐induced increase in PrP^C^ expression (Figure 3F,G). Melatonin also significantly augmented the expression of MnSOD in MSCs (Figure 3H,I). In addition, melatonin increased both catalase and MnSOD activities confirming that melatonin can enhance antioxidant activity (Figure 3J,K). Furthermore, in a murine hindlimb ischaemia model, immunofluorescent staining for MnSOD showed that the expression of MnSOD was increased in transplanted MSCs treated with melatonin (Figure 3L). Importantly, silencing of PrP^C^ in MSCs blocked these anti‐oxidant effects of melatonin (Figure 3H–L and Figure 2). These data suggest that melatonin enhances ROS scavenger enzyme activity via the MT_2_‐mediated upregulation of PrP^C^.

### 3.4 Melatonin attenuated ER stress under ischaemic conditions by regulation of PrP^C^

**Figure 4:**
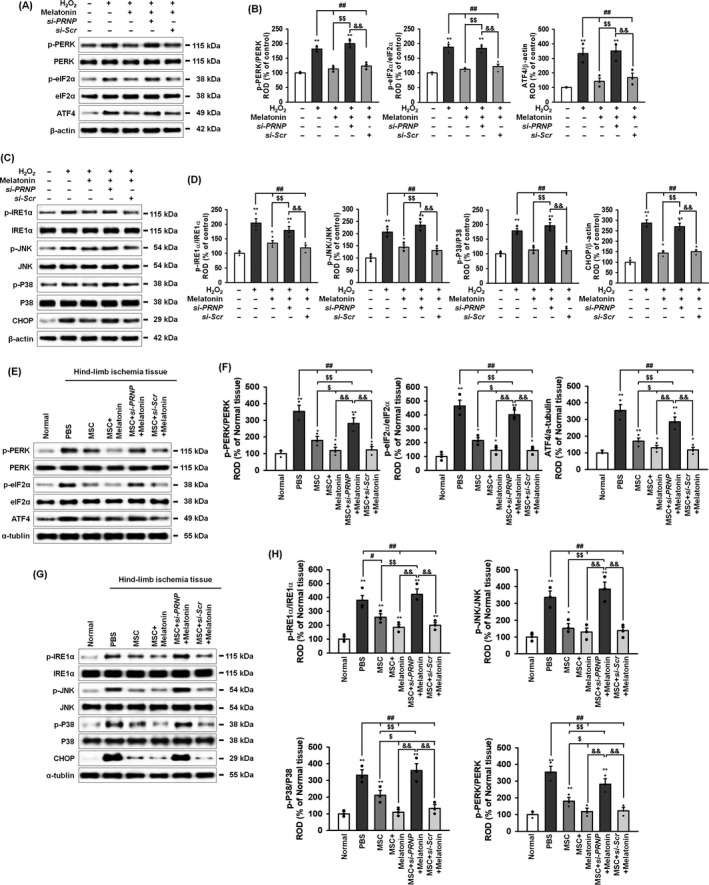
Melatonin protects against oxidative ER stress through the upregulation of PrP^C^. A, Western blot of p‐PERK, PERK, p‐eIF2α, eIF2α and ATF4 after treatment of melatonin‐pretreated MSCs with hydrogen peroxide (200 μmol/L). B, The levels of p‐PERK, p‐eIF2α and ATF4 were normalized to those of PERK, eIF2α and β‐actin, respectively. Values represent the mean ± SEM. *P < 0.05; **P < 0.01 vs untreated MSCs, ^##^ *P* < 0.01 vs MSCs treated with H_2_O_2_, ^$$^ *P* < 0.01 vs MSCs treated with melatonin, and ^&&^ *P* < 0.01 vs MSCs treated with melatonin and *si‐PRNP*. C, Western blot of p‐IRE1α, IRE1α, p‐JNK, JNK, p‐p38, p38 and CHOP after treatment of melatonin‐pretreated MSCs with hydrogen peroxide (200 μmol/L). D, The levels of p‐IRE1α, p‐JNK, p‐p38 and CHOP were normalized to those of IRE1α, JNK, p38 and β‐actin, respectively. Values represent the mean ± SEM. *P < 0.05; **P < 0.01 vs untreated MSCs, ^##^ *P* < 0.01 vs MSCs treated with H_2_O_2_, ^$$^ *P* < 0.01 vs MSCs treated with melatonin, and ^&&^ *P* < 0.01 vs MSCs treated with melatonin and *si‐PRNP*. E, Western blot of p‐PERK, PERK, p‐eIF2α, eIF2α and ATF4 in ischaemic‐injured tissues 1 days after injection with PBS, MSCs (MSC), MSCs treated with melatonin (MSC + Melatonin), MSCs treated with melatonin and *PRNP siRNA* (MSC + si*‐PRNP *+ Melatonin), or MSCs treated with melatonin and scrambled *siRNA* (MSC + si*‐Scr *+ Melatonin) in a murine hindlimb ischaemia model. F, The expressions of p‐PERK, p‐eIF2α and ATF4 were normalized to those of PERK, eIF2α, and α‐tubulin, respectively. Values represent the mean ± SEM. *P < 0.05; **P < 0.01 vs normal, ^##^ *P* < 0.01 vs PBS, ^$^ *P* < 0.05; ^$$^ *P* < 0.01 vs MSC, and ^&&^ *P* < 0.01 vs MSC + si*‐PRNP *+ Melatonin. G, Western blot of p‐IRE1α, IRE1α, p‐JNK, JNK, p‐p38, p38 and CHOP in the same tissues as in E. H, The expression levels of p‐IRE1α, p‐JNK, p‐p38 and CHOP were normalized to those of IRE1α, JNK, p38 and α‐tubulin, respectively. Values represent the mean ± SEM. *P < 0.05; **P < 0.01 vs normal, ^#^ *P* < 0.05; ^##^ *P* < 0.01 vs PBS, ^$^ *P* < 0.05; ^$$^ *P* < 0.01 vs MSC, and ^&&^ *P* < 0.01 vs MSC + si*‐PRNP *+ Melatonin

To elucidate whether melatonin regulates ER stress through PrP^C^ expression, the activation and expression of ER stress–associated proteins were analysed after treatment of MSCs with H_2_O_2_ and melatonin. Melatonin significantly decreased the activation and expression ER stress–associated protein (Figure 4A–D). To further investigate the protective effect of melatonin in vivo, the activation and/or expression levels of these ER stress–associated proteins were also significantly decreased following transplantation with MSCs treated with melatonin (Figure 4E‐H). However, knock‐down of PrP^C^ in the MSCs abolished these inhibitory effects on ER stress–associated proteins. These results suggest that melatonin attenuated ER stress via upregulation of PrP^C^ both in vitro and in vivo.

### 3.5 Melatonin‐induced PrP^C^ protects cells from apoptotic cell death under conditions of ER stress through the regulation of autophagy

**Figure 5:**
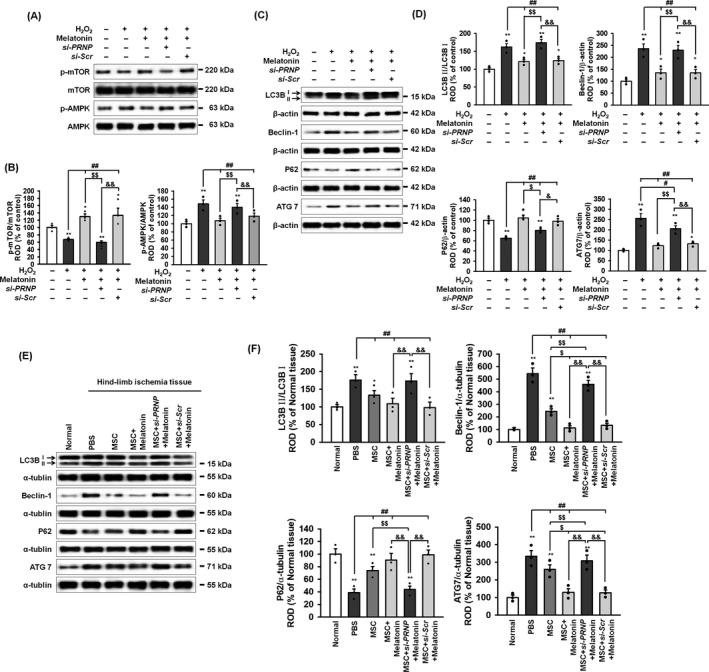
Melatonin inhibits oxidative ER stress–mediated autophagy via PrP^C^ expression. A, Western blot of p‐mTOR, mTOR, p‐AMPK and AMPK after treatment of melatonin‐pretreated MSCs with hydrogen peroxide (200 μmol/L). B, The levels of p‐mTOR and p‐AMPK were normalized to those of mTOR and AMPK, respectively. Values represent the mean ± SEM. **P < 0.05; **P < 0.01 vs untreated MSCs, ^##^ *P* < 0.01 vs MSCs treated with H_2_O_2_, ^$$^ *P* < 0.01 vs MSCs treated with melatonin, and ^&&^ *P* < 0.01 vs MSCs treated with melatonin and *si‐PRNP*. C, Western blot of LC3B, Beclin‐1, p62 and ATG7 after treatment of melatonin‐pretreated MSCs with hydrogen peroxide (200 μmol/L). D, The levels of LC3BII were normalized to LC3B1 whereas the levels of Beclin‐1, p62 and ATG7 were normalized to β‐actin. Values represent the mean ± SEM. *P < 0.05; **P < 0.01 vs untreated MSCs, ^#^ *P* < 0.05; ^##^ *P* < 0.01 vs MSCs treated with H_2_O_2_, ^$^ *P* < 0.05; ^$$^ *P* < 0.01 vs MSCs treated with melatonin, and ^&^ *P* < 0.05; ^&&^ *P* < 0.01 vs MSCs treated with melatonin and *si‐PRNP*. E, Western blot of LC3B, Beclin‐1, p62 and ATG7 in ischaemic‐injured tissues 1 days after injection with PBS, MSCs (MSC), MSCs treated with melatonin (MSC + Melatonin), MSCs treated with melatonin and *PRNP siRNA* (MSC + si*‐PRNP *+ Melatonin), or MSCs treated with melatonin and scrambled *siRNA* (MSC + si*‐Scr *+ Melatonin) in a murine hindlimb ischaemia model. F, The levels of LC3BII were normalized to LC3B1, whereas the levels of Beclin‐1, p62, and ATG7 were normalized to α‐tubulin. Values represent the mean ± SEM. *P < 0.05; **P < 0.01 vs normal, ^##^ *P* < 0.01 vs PBS, ^$^ *P* < 0.05; ^$$^ *P* < 0.01 vs MSC, and ^&&^ *P* < 0.01 vs MSC + si*‐PRNP *+ Melatonin

To explore whether melatonin protects ER stress–mediated autophagy in MSCs and ischaemic tissues through the regulation of PrP^C^, we analysed the expression of autophagy markers in the MSCs and ischaemic tissues transplanted with MSCs. Autophagy is regulated through the mTOR and AMPK signalling pathway.^32^ In MSCs, treated with H_2_O_2_ to induce ROS‐mediated ER stress, melatonin activated mTOR and inhibited AMPK phosphorylation (Figure 5A,B). Silencing of PrP^C^ in the MSCs inhibited these melatonin‐mediated effects on the upstream autophagy signalling pathway. Under this same oxidative stress condition, in MSCs, melatonin suppressed the H_2_O_2_‐mediated induction of the autophagy inducing proteins, LC3B, Beclin‐1 and ATG7, and in parallel increased the expression of the autophagy repressor, p62 (Figure 5C,D). Moreover, in ischaemic‐injured tissues from the murine hindlimb ischaemia model, the levels of autophagy inducer proteins (LC3B, Beclin‐1, and ATG7) were decreased and the level of the autophagy repressor protein p62 was increased following transplantation with MSCs treated with melatonin (Figure 5E,F). These protective effects of melatonin on autophagy were all significantly ablated by silencing of PrP^C^, both in vitro and in vivo. These findings suggest that melatonin‐induced PrP^C^ protected autophagy under oxidative ER‐stress condition through regulation of the mTOR‐AMPK axis.

**Figure 6:**
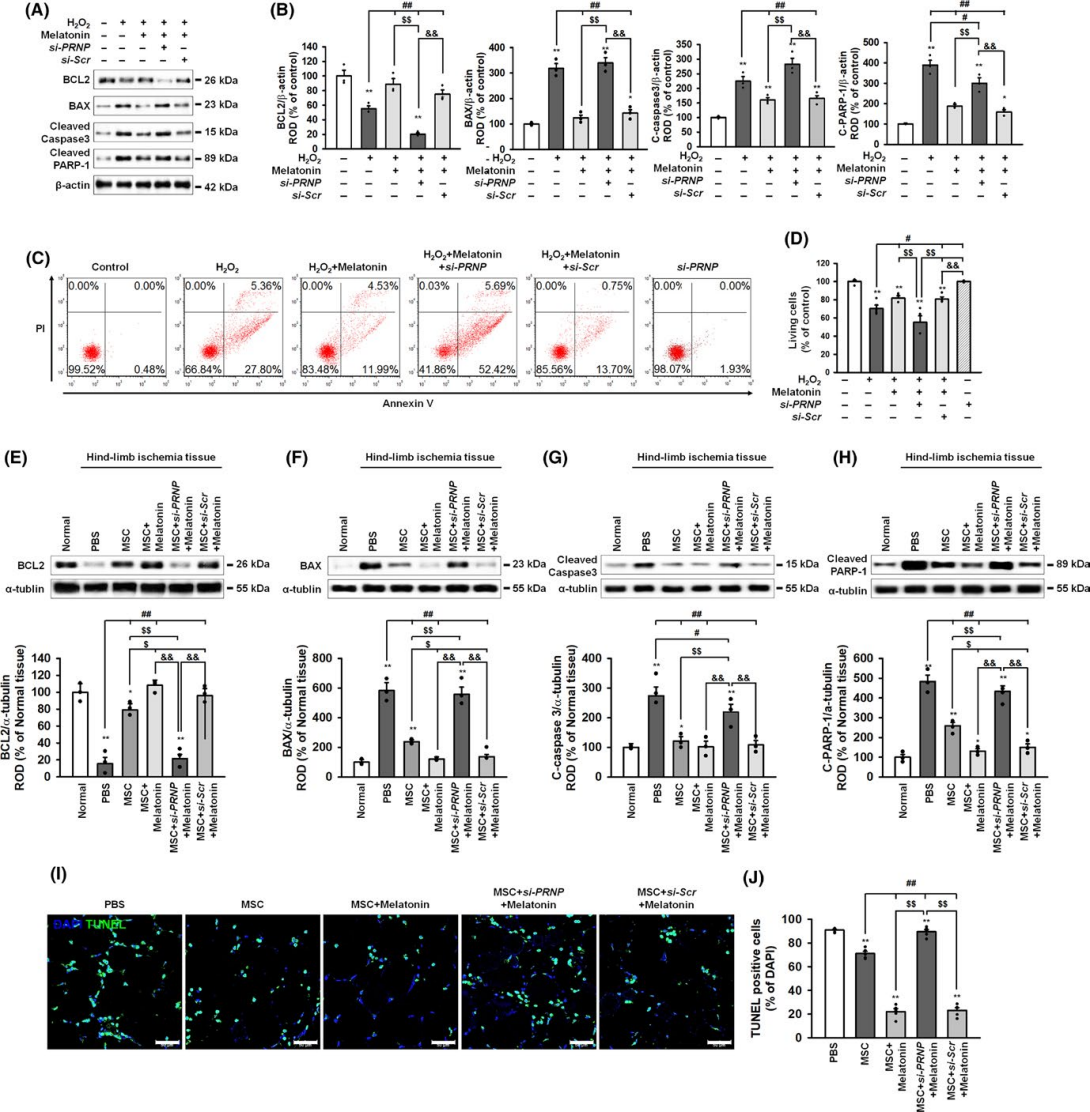
Melatonin protects against oxidative ER stress–mediated cell death via upregulation of PrP^C^. A, Western blot of BCL2, BAX, cleaved caspase‐3 and cleaved PARP after treatment of melatonin‐pretreated MSCs with hydrogen peroxide (200 μmol/L). B, The expressions of these proteins were normalized to that of β‐actin. Values represent the mean ± SEM. *P < 0.05; **P < 0.01 vs untreated MSCs, ^##^ *P* < 0.01 vs MSCs treated with H_2_O_2_, ^$$^ *P* < 0.01 vs MSCs treated with melatonin, and ^&&^ *P* < 0.01 vs MSCs treated with melatonin and *si‐PRNP*. C, Flow cytometry analysis of PI and Annexin V staining after treatment of melatonin‐pretreated MSCs with H_2_O_2_. D, The number of surviving cells was quantified as the percentage of PI and Annexin V negative cells. Values represent the mean ± SEM. **P < 0.01 vs untreated MSCs, ^#^ *P* < 0.05 vs MSCs treated with H_2_O_2_, and ^$$^ *P* < 0.01 vs MSCs treated with melatonin and *si‐PRNP*, and ^&&^ *P* < 0.01 vs MSCs treated with *si‐PRNP*. (E‐H) Western blot of BCL2, BAX, cleaved caspase‐3 and cleaved PARP ischaemic‐injured tissues 1 days after injection with PBS, MSCs (MSC), MSCs treated with melatonin (MSC + Melatonin), MSCs treated with melatonin and *PRNP siRNA* (MSC + si*‐PRNP *+ Melatonin), or MSCs treated with melatonin and scrambled *siRNA* (MSC + si*‐Scr *+ Melatonin) in a murine hindlimb ischaemia model. The expression levels of BCL2, BAX, cleaved caspase‐3 and cleaved PARP were normalized to that of α‐tubulin. Values represent the mean ± SEM. *P < 0.05; **P < 0.01 vs normal, ^#^ *P* < 0.05; ^##^ *P* < 0.01 vs PBS, ^$^ *P* < 0.05; ^$$^ *P* < 0.01 vs MSC, and ^&&^ *P* < 0.01 vs MSC + si*‐PRNP *+ Melatonin. I, TUNEL assay in ischaemic‐injured tissues from the groups in E‐H. Scale bar = 50 μm. J, Apoptotic cells were quantified as the percentage of TUNEL (green)‐positive cells per total DAPI‐stained cells. Values represent the mean ± SEM. *P < 0.05; **P < 0.01 vs PBS, ^#^ *P* < 0.05 vs MSC, ^$^ *P* < 0.05; ^$$^ *P* < 0.01 vs MSC + Melatonin

To assess whether melatonin protects against apoptotic cell death under conditions of ER stress, through the upregulation of PrP^C^ expression, the level of apoptosis‐associated proteins and cell death were assessed in MSCs and in ischaemic tissues transplanted with MSCs. In MSCs treated with H_2_O_2_ to induce ROS‐mediated ER stress, melatonin reduced apoptosis pathway (Figure 6A,B). Moreover, a PI‐Annexin V staining assay of MSCs showed that melatonin protected against ROS‐mediated apoptotic cell death (Figure 6C,D). In the hindlimb ischaemia, transplantation with MSCs treated with melatonin reduced expression of apoptosis protein (Figure 6E‐H). In addition, a TUNEL assay of the ischaemically injured tissues showed that ischaemia‐induced, ER stress–mediated, cellular apoptosis was significantly decreased following transplantation of MSCs treated with melatonin, compared with other MSC‐treated groups (Figure 6I,J). These protective effects of melatonin on apoptotic cell death under oxidative ER‐stress conditions, both in vitro and in vivo, were reversed by silencing of PrP^C^ in the MSCs. Our findings suggest that the melatonin‐induced upregulation of PrP^C^ protects against apoptotic cell death under oxidative ER‐stress conditions through regulation of the autophagy process.

### 3.6 Melatonin‐treated MSCs enhance neovascularization in a murine hindlimb ischaemia model

**Figure 7:**
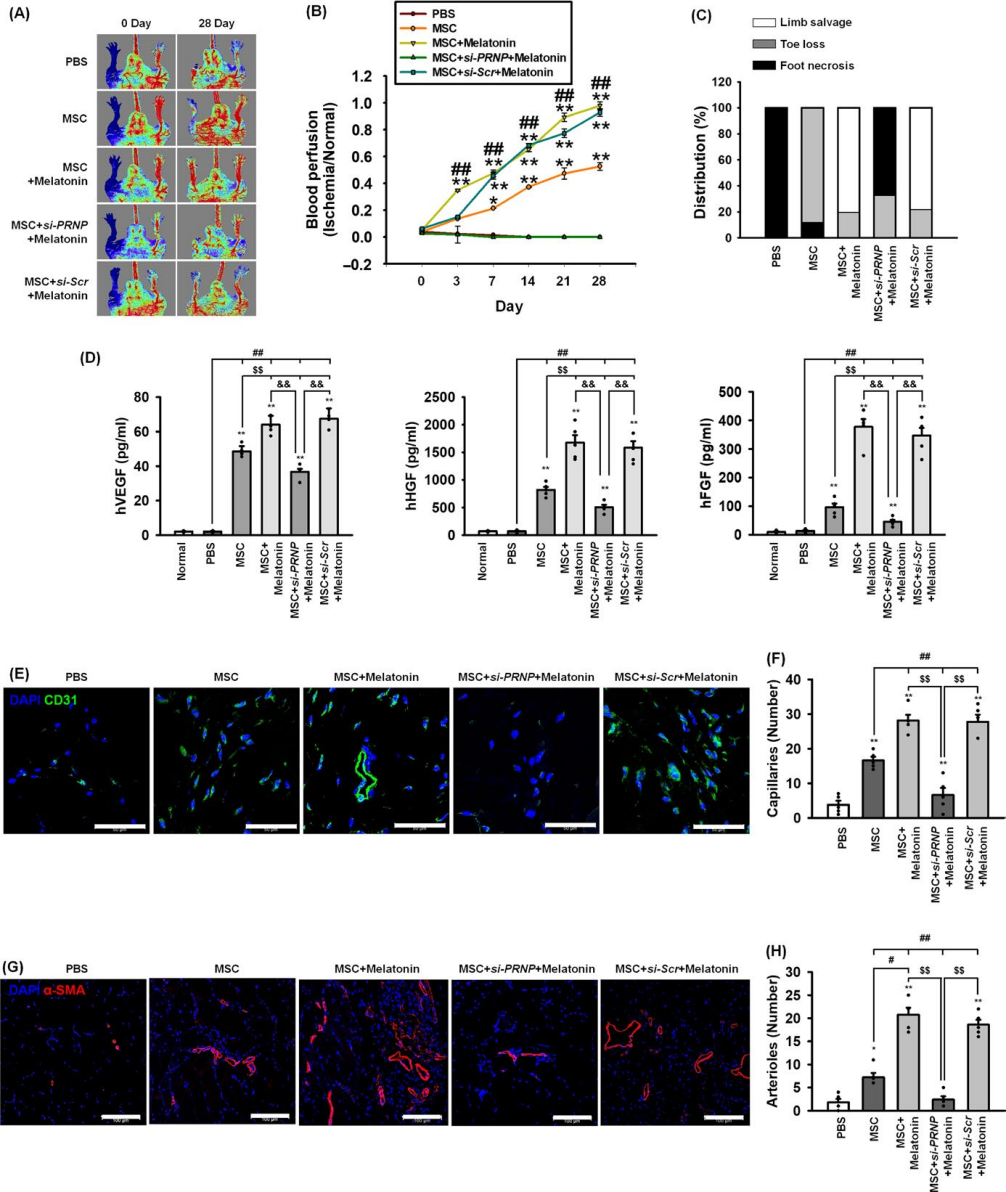
Assessment of functional recovery and neovascularization in a murine hindlimb ischaemia model. A, Blood perfusion was assessed using laser Doppler perfusion imaging analysis in the ischaemic limbs of mice injected with PBS, MSCs (MSC), melatonin‐treated MSCs (MSC + Melatonin), MSCs treated with melatonin and *PRNP siRNA* (MSC + si*‐PRNP *+ Melatonin), or MSCs treated with melatonin and scrambled *siRNA* (MSC + Scr*‐siRNA *+ Melatonin). B, The blood perfusion ratio was measured by LPDI analysis (blood flow in the left ischaemic limb/blood flow in the right non‐ischaemic limb). Values represent the mean ± SEM. *P < 0.05; **P < 0.01 vs PBS, and ^##^ *P* < 0.01 vs MSC. C, Distribution of the different outcomes (limb salvage, toe loss and foot necrosis) in each of the treatment groups at post‐operative day 28. D, Secretion of human VEGF, HGF and FGF from the ischaemic‐injured tissues in each group was assessed by ELISA. Values represent the mean ± SEM. **P < 0.01 vs normal, ^##^ *P* < 0.01 vs PBS, ^$$^ *P* < 0.01 vs MSC, and ^&&^ *P* < 0.01 vs MSC + si*‐PRNP *+ Melatonin. E‐G At post‐operative day 28, the formation of capillaries and arterioles were analysed using immunofluorescent staining for CD31 (green; scale bar = 50 μm; E) and α‐SMA (red; scale bar = 100 μm; G). The capillary (F) and arteriole (H) densities were quantified as the number of CD31‐positive cells and α‐SMA‐positive cells, respectively. Values represent the mean ± SEM. *P < 0.05; **P < 0.01 vs PBS, ^#^ *P* < 0.05; ^##^ *P* < 0.01 vs MSC, ^$$^ *P* < 0.01 vs MSC+si*‐PRNP *+ Melatonin

To examine whether melatonin‐treated MSCs could promote the formation of neovessels in a murine hindlimb ischaemia model, blood perfusion and tissue recovery were assessed following transplantation of MSCs into the ischaemic‐injured tissue. To stimulate the transplanted MSCs over the long‐term, an intraperitoneal injection of melatonin was performed every day for 28 days following surgery. The blood perfusion ratio was analysed using LDPI at post‐operative day 0, 3, 7, 14, 21 and 28. The blood perfusion ratio was significantly higher in limbs transplanted and maintained with the melatonin‐treated MSCs than with any other group (Figure 7A,B). In addition, the percentage of limb salvaged was significantly higher in limbs transplanted and maintained with the melatonin‐treated MSCs than with any other group (Figure 7C). In ischaemic‐injured tissues, the secretion of the angiogenic cytokines, such as human VEGF, HGF and FGF, was significantly higher in mice transplanted and maintained with the melatonin‐treated MSCs than with any other group (Figure 7D). Immunofluorescent staining for CD31 (capillary) and α‐SMA (arterioles) revealed that the densities of capillaries and arterioles were significantly increased in mice transplanted and maintained with the melatonin‐treated MSCs than with any other group (Figure 7E‐H). Inhibition of PrP^C^ expression significantly prevented these beneficial effects of melatonin on neovascularization. However, the densities of capillaries and arterioles at the sites injected with melatonin‐treated MSCs transfected with *PRNP* siRNA were significantly lower than those at the sites injected with MSCs alone. This is the reason for the significant increase in the level of pro‐apoptotic proteins, including BAX, cleaved caspase‐3 and cleaved PARP‐1, at sites injected with melatonin‐treated MSCs transfected with *PRNP* siRNA compared with that at the sites injected with MSCs alone (see, Figure 6F‐H); this effect also led to the increased apoptosis in ischaemic‐injured tissues (see, Figure 6I,J). In addition, oxidative stress increased the expression PrP^C^ (see, Figure 3A). Therefore, *PRNP* siRNA might suppress the physiological increase in PrP^C^, thereby reducing the functional recovery at sites injected with melatonin‐treated MSCs transfected with *PRNP* siRNA compared with that at the sites injected with MSCs alone. These results indicate that transplantation with MSCs stimulated with melatonin improves neovascularization and functional recovery through the expression of PrP^C^.

## 4 DISCUSSION

Ischaemic‐injured sites have harsh tissue conditions, including low oxygen levels, restricted nutrients, and high levels of ROS, with the result that effectiveness of the transplanted stem/progenitor cells used for regenerative medicine to treat ischaemic diseases is decreased. Among the pathophysiological conditions, ischaemia/ROS‐induced ER stress is known to induce apoptotic cell death in host tissues as well as in the transplanted stem/progenitor cells.^6^, ^7^, ^17^, ^29^ To solve this issue, several studies have suggested that melatonin could be used to protect against ER stress in neurotoxic states, liver diseases, immunotoxic states, pulmonary fibrosis and diabetes.^33^ Our previous study revealed that melatonin could enhance MSC functionality for the treatment of ischaemic disease by upregulating the express of PrP^C^.^16^ To further examine whether melatonin protects against ER stress through the upregulation of PrP^C^, we have confirmed the protective effect of melatonin on autophagy‐mediated cell death in MSCs with ischaemia‐induced ER stress. Our results showed that melatonin‐induced PrP^C^ expression decreased ER stress/autophagy‐mediated apoptosis both in vitro and in a murine hindlimb ischaemia model.

Various reports have indicated that PrP^C^, which is expressed in many cells beyond the central nerve system,^34^ contributes to the regulation of several cellular process, including differentiation, proliferation, stress protection, myelin maintenance, mitochondrial homoeostasis and interaction with signal transduction pathways.^11^, ^35^ In particular, PrP^C^ has been shown to have a protective effect against ROS‐mediated oxidative stress.^11^ PrP^C^ suppresses ROS production by regulating the activities of superoxide dismutase and glutathione peroxidase.^36^, ^37^ In fact, our previous study revealed that TUDCA‐induced PrP^C^ increased MnSOD expression via phosphorylation of Akt and activation of its signalling pathway.^17^ In keeping with the concept of PrP^C^ regulating the activity of enzymes that control ROS levels it has been shown in a PrP‐null mouse, that total SOD activity was significantly decreased through modulation of mitochondrial complex I.^38^ Since ischaemic‐injured tissues represent a ROS‐rich oxidative stress environment, reductions in ROS levels are important for the successful transplantation of stem/progenitor cells in cell‐based therapies. One study has shown that PrP^C^ enhances MSC engraftment to bone marrow.^39^ Hypoxia‐induced PrP^C^ expression has also been shown to facilitate the survival of transplanted MSCs at ischaemic‐damaged sites in hindlimb model of ischaemia.^15^ Our data indicated that melatonin increased the activation of MnSOD and catalase activity in MSCs under ROS‐mediated oxidative stress through the upregulation of PrP^C^. Moreover, the expression of MnSOD in transplanted MSCs treated with melatonin was augmented in the ischaemic‐injured sites, resulting in the suppression of apoptosis.

Melatonin is known to be a powerful ROS scavenger through this regulation of antioxidant enzymes.^40^, ^41^ Several studies have investigated the protective effect of melatonin in MSCs with ischaemic injury in order to increase their efficacy in transplantation. In small bowel ischaemia‐reperfusion injury, injection with melatonin, along with adipose‐derived MSCs, decreased ischaemic damage through the regulation of antioxidant enzymes, notably NAD(P)H dehydrogenase, glutathione reductase and glutathione peroxidase.^42^ In addition, melatonin blocked oxidative stress‐induced premature senescence and promoted the therapeutic efficacy of MSCs in a murine myocardial infarction model via a sirtuin‐1‐dependent mechanism.^24^, ^43^ Our results therefore confirmed that melatonin stimulation of MSCs enhances the functional recovery and neovascularization in a murine hindlimb ischaemia model via PrP^C^ expression. Consistent with these results, our previous studies have shown that PrP^C^ is a key molecule in regulating the functionality and therapeutic potential of MSCs in ischaemic diseases.^15^, ^16^, ^17^ Taken together, our data show that melatonin protects against ROS‐mediated oxidative stress by regulating PrP^C^‐dependent antioxidant enzyme activity.

This study also showed that ROS/ischaemia triggered the activation of ER stress–associated proteins in both MSCs in vitro, and in ischaemic‐injured tissues in vivo. Prolonged activation of PERK and IRE1α induce apoptotic cell death under pathophysiological conditions.^44^ In addition to activation of the unfolded protein response ER stress stimulates autophagy. Autophagy is cellular process that is essential for cell survival, development, differentiation and cellular homoeostasis.^45^ Under physiological conditions, the basal level of autophagy plays an important role in cellular adaptation to nutrient starvation, endo‐lysosomal degradation, the removal of misfolded and long‐lived proteins, and other cellular stresses caused by detrimental cellular substances and invading pathogens.^8^ However, excessive autophagy, initiated by long‐term stress, triggers cellular damage, resulting in the induction of apoptotic cell death.^8^, ^46^ However, some studies have suggested that inhibition of autophagy protects against ischaemic‐induced injury, whereas other studies indicate that activation of autophagy inhibits apoptosis.^33^, ^46^, ^47^ Our data indicated that melatonin protected against ischaemia‐induced ER stress in both MSCs and in a murine hindlimb ischaemia model. Treatment of MSCs with melatonin increased antioxidant effects, thereby increasing the survival of transplanted cells. In addition, melatonin‐treated MSCs increased the secretion of angiogenic cytokines, such as VEGF, HGF and FGF, in ischaemic‐injured tissues, thereby increasing neovascularization. Of particular interest is that these protective effects were regulated by melatonin‐mediated PrP^C^ upregulation. Several other studies have documented that melatonin can inhibit ischaemia‐induced ER stress. For example, treatment melatonin restored hypoxic‐ischaemic encephalopathy and ischaemia‐reperfusion injury in liver through reduced ER stress activity.^48^, ^49^ In addition, melatonin treatment has been reported to attenuate ischaemia/reperfusion‐induced ER stress by regulating the PERK and IRE1 pathways.^46^ This study showed melatonin protected against ER stress/autophagy‐mediated apoptotic cell death by appropriately regulating the expression of pro‐ and anti‐apoptotic proteins. Of particular importance, these beneficial effects were blocked by silencing of PrP^C^. These results are in good agreement with our previous findings showing that melatonin regulated apoptosis‐associated proteins under oxidative stress conditions by upregulating PrP^C^.^16^ In the liver of leptin‐deficient mice, melatonin has also been shown to inhibit oxidative damage through a reduction in ER stress and autophagy.^50^

**Figure 8:**
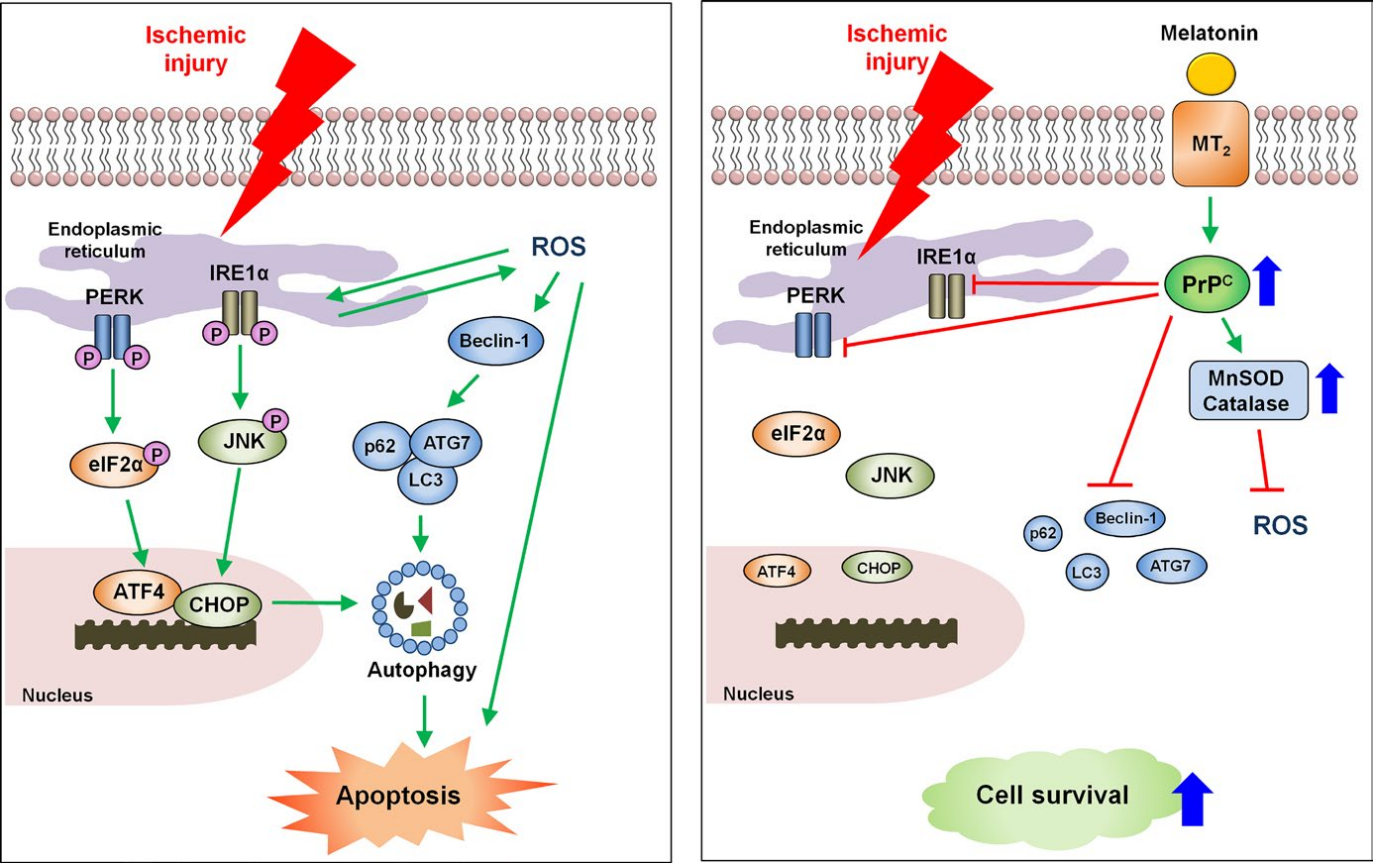
Schematic representation of the proposed mechanisms by which melatonin protects against ER stress/autophagy‐induced cell death through upregulation of the cellular prion protein (PrP^C^) levels. Treatment of MSCs with melatonin inhibits ER stress, autophagy and apoptosis via PrP^C^ upregulation. Moreover, melatonin increases the activation of MnSOD and catalase activity. Transplantation of melatonin‐treated MSCs improves the functional recovery and vessel formation in ischaemic diseases through this melatonin‐mediated PrP^C^ expression

This study therefore suggests that melatonin‐stimulated PrP^C^ protects against ischaemic injury and apoptotic cell death by inhibiting the ER stress‐autophagy axis both in vitro and in vivo. Of particular note, we demonstrated for the first time that PrP^C^ is involved in melatonin‐mediated ER stress and autophagy inhibition both in MSCs and in a hindlimb ischaemia model (Figure 8). Our findings therefore indicate that melatonin is a key regulator of ER stress and autophagy. In addition, our results suggest that melatonin‐induced PrP^C^ is a crucial protective molecule for cell survival against apoptosis induced by ER stress–mediated autophagy. These observations provide important insights into other ischaemia‐induced cell death mechanisms that are inhibited by melatonin‐induced PrP^C^ expression.

## CONFLICT OF INTEREST

The authors declare no conflict of interests.

## AUTHOR CONTRIBUTIONS

JHL: study concept and design, acquisition of data, analysis and interpretation of data, drafting of the manuscript. YMY, YSH and SKJ: acquisition of data, analysis and interpretation of data, statistical analysis. SHL: study concept and design, acquisition of data, analysis and interpretation of data, drafting of the manuscript, procurement of funding, study supervision.
