# Supplementary figures and images for: Melatonin protects mesenchymal stem cells from autophagy‐mediated death under ischaemic ER‐stress conditions by increasing prion protein expression
Source: Cell Prolif. 2018 Nov 14;52(2):e12545. doi: 10.1111/cpr.12545 (PMC6495509; doi:10.1111/cpr.12545)

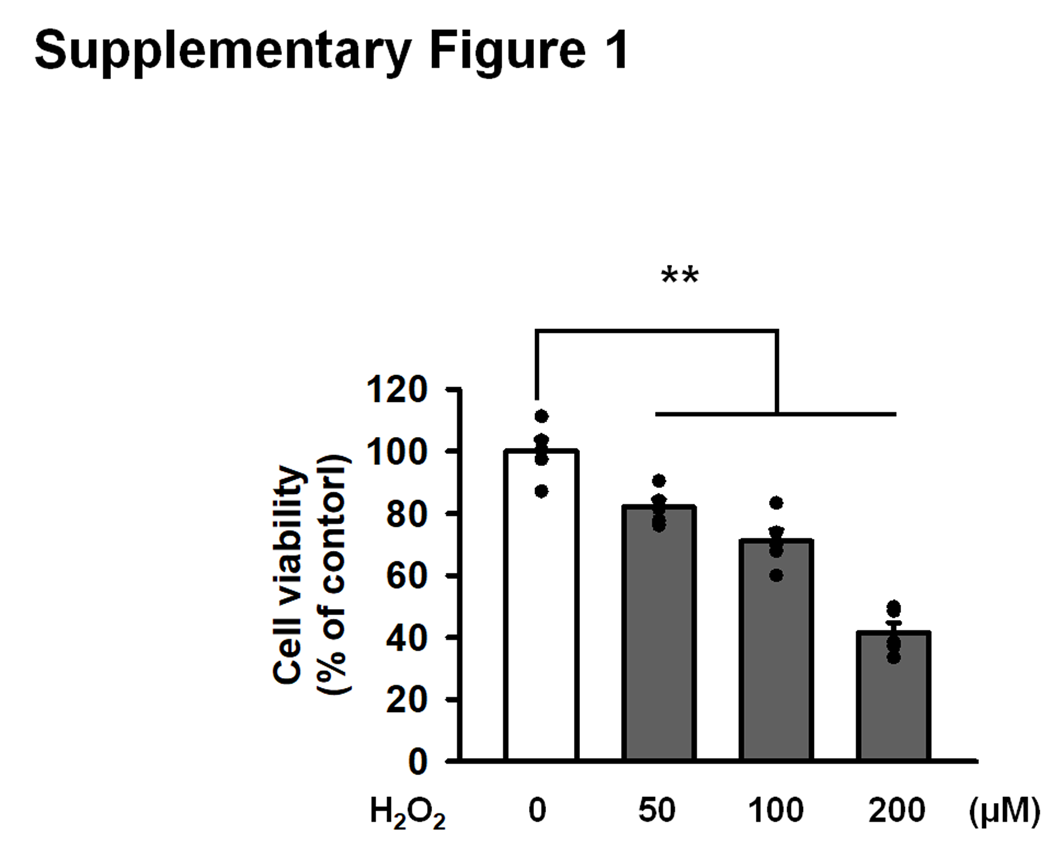

Supplement: Supplementary file 1 [file CPR-52-e12545-s001.tif]

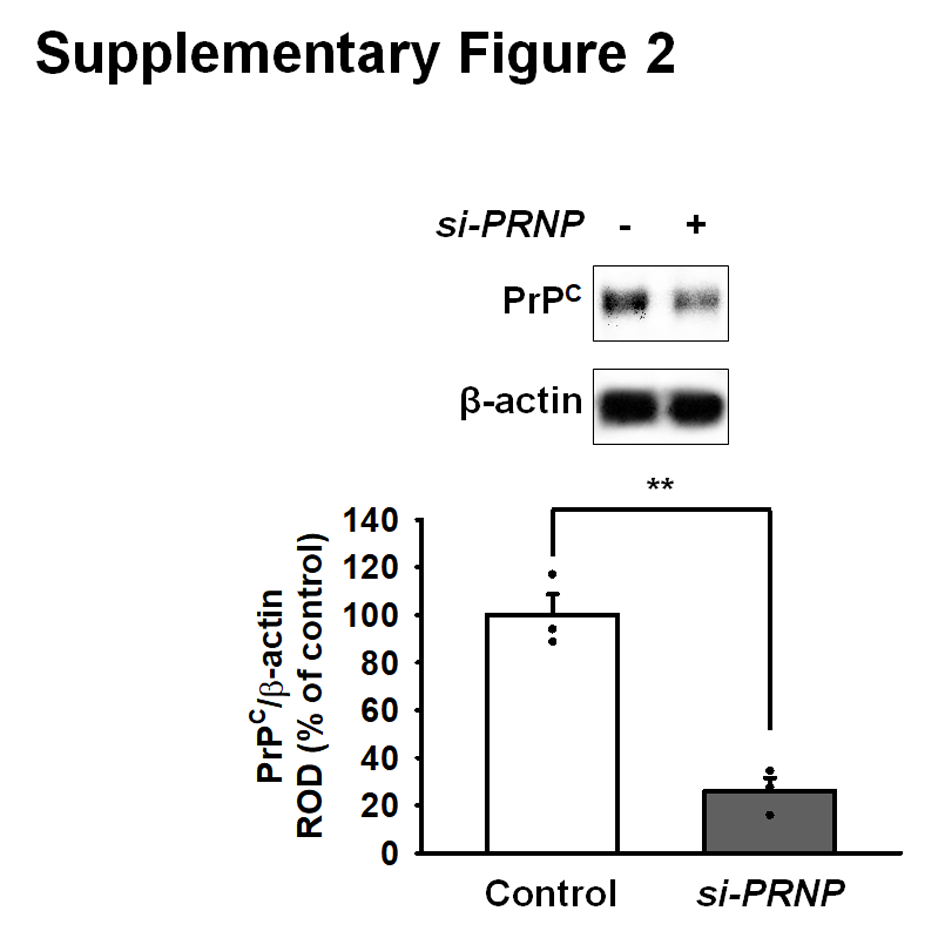

Supplement: Supplementary file 2 [file CPR-52-e12545-s002.tif]
