# Supplementary material for: Melatonin protects mesenchymal stem cells from autophagy‐mediated death under ischaemic ER‐stress conditions by increasing prion protein expression
Source: Cell Prolif. 2018 Nov 14;52(2):e12545. doi: 10.1111/cpr.12545 (PMC6495509; doi:10.1111/cpr.12545)
Supplement: Supplementary file 3 [file CPR-52-e12545-s003.docx]

**Supplemental Figure Legends**

**Supplemental Figure 1.** Effect of H_2_O_2_ on the viability of MSCs.

Viability of MSCs was assayed after treatment with H_2_O_2_ (0, 50, 100, and 200 µM). Values represent the mean ± SEM. **P* < 0.05; ***P* < 0.01 vs. untreated MSCs.

**Supplemental Figure 2.** Knockdown of *PRNP* in MSCs.

MSCs were transfected with *PRNP* siRNA (*si-PRNP*), and the level of PrP^C^ was assessed by western blot. PrP^C^ was quantified by densitometry and normalized to the that of β-actin. Values represent the mean ± SEM. ***P* < 0.01.
