# Supplementary material for: Melatonin protects mesenchymal stem cells from autophagy‐mediated death under ischaemic ER‐stress conditions by increasing prion protein expression
Source: Cell Prolif. 2018 Nov 14;52(2):e12545. doi: 10.1111/cpr.12545 (PMC6495509; doi:10.1111/cpr.12545)
Supplement: Supplementary file 4 [file CPR-52-e12545-s004.docx]

**Appendix S1. Material & Methods**

**1.1. Western blot analysis**

Mesenchymal stem cell (MSC) homogenates (20 µg protein) were separated by 8–12% sodium dodecyl sulfate-polyacrylamide gel electrophoresis, and the bands were transferred to polyvinylidene fluoride membranes (Sigma, St. Louis, MO, USA). The membranes were blocked with 5% skimmed milk for 1 h and incubated with primary antibodies against protein kinase R-like endoplasmic reticulum kinase (PERK), phospho-PERK, eukaryotic initiation factor 2-alpha (eIF2α), phospho-eIF2α, activating transcription factor 4 (ATF4), inositol-requiring protein 1α (IRE1α), phospho-IRE1α, c-Jun N-terminal kinase (JNK), phospho-JNK, CCAAT-enhancer-binding protein homologous protein (CHOP), B-cell lymphoma 2 (BCL-2), BCL-2-associated X protein (BAX), cleaved caspase-3, cleaved poly(ADP ribose) polymerase-1 (PARP-1), microtubule-associated proteins 1A/1B light chain 3 (LC3), Beclin-1, p62, autophagy-related protein 7 (ATG7), PrP^C^, manganese-dependent superoxide dismutase (MnSOD), p-38, phospho-p38, mechanistic target of rapamycin (mTOR), phospho-mTOR, 5’ adenosine monophosphate-activated protein kinase (AMPK), phospho-AMPK, α-tubulin, and β-actin. All antibodies were purchased from Santa Cruz Biotechnology (Santa Cruz, CA, USA). The membranes were then washed, and the primary antibodies were detected using goat anti-rabbit IgG or goat anti-mouse IgG conjugated to horseradish peroxidase (Santa Cruz). The bands were then visualized using enhanced chemiluminescence (Sigma).

**1.2. Inhibition of cellular prion protein expression by RNA interference.**

MSCs (2 × 10^5^) were seeded in 60-mm dishes and transfected with siRNA in serum-free Opti-MEM (Thermo Fisher Scientific) using Lipofectamine 2000, according to the manufacturer’s instructions. After transfection for 48 h, total protein was extracted and protein expression was determined by western blot analysis. The siRNA targeting PRNP and the scrambled control sequence were synthesized by Bioneer (Daejeon, Korea).

**1.3. Immunohistochemistry**

At 1, 3, and 28 days post-operation, the ischemic tissues were removed and fixed with 4% paraformaldehyde (Sigma). Each tissue sample was embedded in paraffin. Immunofluorescence staining was performed using primary antibodies against MnSOD, PrP^C^, CD31, and α-SMA (Santa Cruz Biotechnology) and secondary antibodies Alexa-488 and Alexa-594, as appropriate (Thermo Fisher Scientific). Nuclei were stained with 4′,6-diaminido-2-phenylindol (DAPI; Sigma), and the immunostained samples were observed using confocal microscopy (Olympus).

**1.4. Catalase activity**

Prior to the measurement of enzyme activity, MSCs were plated in 100-mm tissue culture plates and grown to 70–75% confluence. Cells were pretreated with melatonin (1 μM) for 24 h and then collected and resuspended in lysis buffer (1% Triton X-100 in 50 mM Tris-HCl [pH 7.4]) containing 150 mM NaCl, 5 mM EDTA, 2 mM Na_3_VO_4_, 2.5 mM Na_4_PO_7_, 100 mM NaF, and protease inhibitors. Samples were incubated for 30 min on ice and then centrifuged at 14000 rpm for 30 min at 4°C. The protein concentration of the supernatant was determined using the Micro BCA assay (Thermo Fisher Scientific). The Catalase Assay Kit (Sigma) was used to measure catalase activity using H_2_O_2_ (200 mM) as the substrate and by recording the decrease in absorbance at 240 nm. Catalase activity was expressed in mU/mg protein.

**1.5. Measurement of SOD activity**

MSCs were pretreated with melatonin (1 μM) for 24 h and then harvested from the culture plate. The protein concentration of cell lysates was determined using the bicinchoninic acid assay (Sigma). The SOD activity assay is based on the reduction of water-soluble tetrazolium by superoxide anion (produced from xanthine by xanthine oxidase) to produce a colored water-soluble tetrazolium formazan product. Changes in absorbance at 480 nm were recorded using a microplate reader (BMG Labtech, Ortenberg, Germany).

**1.6.** Terminal deoxynucleotidyl transferase-mediated dUTP nick end labeling (TUNEL) **assay**

The TUNEL assay was performed using a TdT Fluorescein *In Situ* Apoptosis Detection Kit (Trevigen, Inc, Gaithersburg, MD, USA). At postoperative day 3, the assay was performed on tissues obtained from the ischemically injured thigh sites. Stained sections were visualized using a confocal microscope (Olympus).
